# Cold Applications and Sulph. of Copper in Croup

**Published:** 1858-12

**Authors:** 


### COLD APPLICATIONS AND SULPH. OF COPPER IN CROUP.

Dr. Pudon relates some cases as examples of the great benefit he has derived from the continuous application of cold, wet compresses to the neck, simultaneously with the administration of sulph. copper in two grain doses, every half hour; sixty-four grains having been given in one case, and seventy in another.—*Journal fur Kinder: Med. Times and Gazette.*

